# Supplementary material for: Enhanced Biodegradation of Phenylurea Herbicides by Ochrobactrum anthrophi CD3 Assessment of Its Feasibility in Diuron-Contaminated Soils
Source: Int J Environ Res Public Health. 2022 Jan 26;19(3):1365. doi: 10.3390/ijerph19031365 (PMC8835586; doi:10.3390/ijerph19031365)
Supplement: Supplementary file 1 [file ijerph-19-01365-s001.zip › ijerph-1555309-supplementary.pdf]

SUPPLEMENTARY DATA

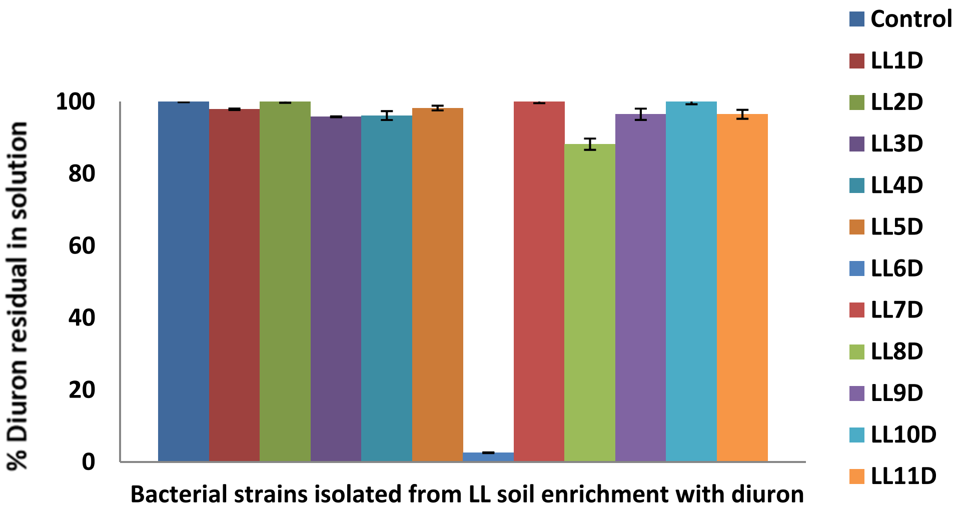

Figure S1. Quantification of the diuron degraded in aqueous solution after 20 days by the bacterial strains isolated from an agricultural soil.

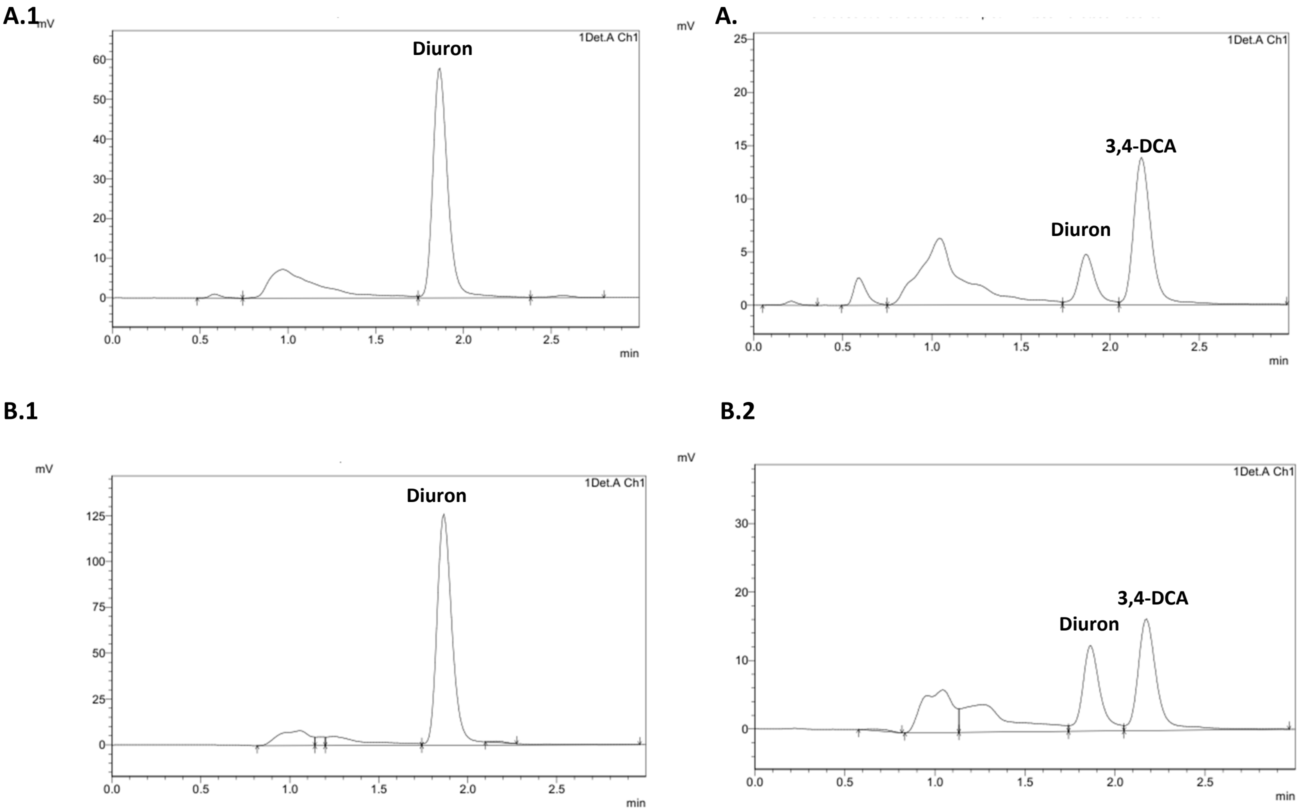

Figure S2 HPLC chromatograms obtained from the extracts of PLD soil (A.1) and R soil (B.1) after one day of incubation; and from PLD soil (A.2) and R soil (B.2) after eight days of incubation.

Table S1. Formulas, molecular structures and some properties of phenylurea herbicides studied in this work (PPDB. IUPAC Pesticides Properties DataBase).

Name	Molecular Structure	Water solubility (mg L^{-1})	Log K_{ow}	K_{oc}	Soil degradation DT_{50}**	Environmental fate
**Linuron** 3-(3,4-dichlorophenyl)-1-methoxy-1-methylurea	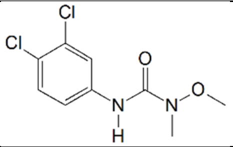	63.8	3.0	843	48 / 57.6	Moderately persistent/ Slightly mobile
**Diuron** 3-(3,4-dichlorophenyl)-1,1-dimethylurea	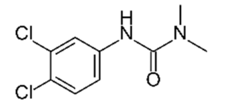	35.6	2.87	680	146.6 / 229	Persistent
**Chlorotoluron** 3-(3-chloro-p-tolyl)-1,1-dimethylurea	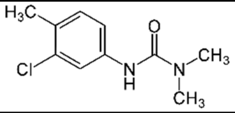	74	2.5	196	-	-
**Isoproturon** 3-(4-isopropylphenyl)-1,1-dimethylurea	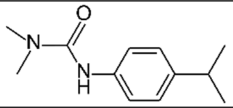	70.2	2.5	122*	12 / 23	Non persistent/ Moderately mobile
**Fluometuron** 1,1-dimethyl-3-(α,α,α-trifluoro-m-tolyl)urea	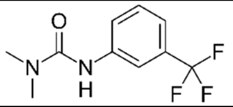	111	2.28	67.4*	63.6 / 89.8	Moderately persistent/Mobile

***Kf_{OC}; **DT_{50}: estimated average half-life in soil (lab at 20 °C / field).**
